# Synthesis and Photoluminescent Properties of Geometrically Hindered *cis*-Tris(diphenylaminofluorene) as Precursors to Light-Emitting Devices

**DOI:** 10.3390/molecules20034635

**Published:** 2015-03-13

**Authors:** Nam-Goo Kang, Ken Kokubo, Seaho Jeon, Min Wang, Chang-Lyoul Lee, Taizoon Canteenwala, Loon-Seng Tan, Long Y. Chiang

**Affiliations:** 1 Department of Chemistry, Institute of Nanoscience and Engineering Technology, University of Massachusetts Lowell, Lowell, MA 01854, USA; E-Mails: papyrus92@gmail.com (N.-G.K.); seaho_jeon@uml.edu (S.J.); wangmin81@gmail.com (M.W.); taizoonc@hotmail.com (T.C.); 2 Division of Applied Chemistry, Graduate School of Engineering, Osaka University, Suita, Osaka 565-0871, Japan; E-Mail: kokubo@chem.eng.osaka-u.ac.jp; 3 Advanced Photonics Research Institute, Gwangju Institute of Science and Technology, Gwangju 500–712, Korea; E-Mail: vsepr@gist.ac.kr; 4 AFRL/RXBN, Air Force Research Laboratory, Wright-Patterson Air Force Base, Dayton, OH 45433, USA; E-Mail: Loon-Seng.Tan@wpafb.af.mil

**Keywords:** *cis*-tris(diphenylaminofluorene), light-harvesting nanostructure, light emitting chromophore, geometrically hindered diphenylaminofluorene, nonplanar stereoisomer

## Abstract

A novel highly luminescent tris-fluorenyl ring-interconnected chromophore tris(DPAF-C_9_) was synthesized using a *C*_3_ symmetrical triaminobenzene core as the synthon. This structure bears three light-harvesting 2-diphenylamino-9,9-dialkylfluorenyl (DPAF) ring moieties with each attached by two branched 3',5',5'-trimethylhexyl (C_9_) arms. A major stereoisomer was chromatographically isolated and characterized to possess a 3D structural configuration of *cis*-conformer in a *cup*-form. Molecular calculation at B3LYP/6-31G* level revealed the unexpected stability of this *cis*-*cup*-conformer of tris(DPAF-C_9_) better than that of the stereoisomer in a *propeller*-form and the *trans*-conformer. The structural geometry is proposed to be capable of minimizing the aggregation related self-quenching effect in the condensed phase. Fluorescence emission wavelength of tris(DPAF-C_9_) was found to be in a close range to that of PVK that led to its potential uses as the secondary blue hole-transporting material for enhancing the device property toward the modulation of PLED performance.

## 1. Introduction

Organic donor fluorophores having extended polarizable ring-branching conjugations in their structure often exhibit good efficiency in photoluminescence that makes them suitable as active components in a number of materials applications, such as optoelectronic devices [1], light-emitting diode (OLED/PLED) cells for flat-panel display [2,3,4], nonlinear optical (NLO) materials for optical limiting (OL) [5], 3D nano/microfabrication [6], one-photon (1γ) or two-photon (2γ) fluorescence-based optical bio-imaging and labeling substrates for medical diagnosis [7,8], biosensors [9,10,11], and photo-diagnosis/photodynamic sensitizers for PDT treatments [12,13]. The former four applications involve thin-film device or coating fabrication processes in a solid form of the material. Therefore, it is crucial for organic donor fluorophores to exhibit either an ultrafast photoresponsive excited state population (for NLO/OL) or intense electroluminescence (for OLED/PLED) in the solid state to increase the performance efficiency. However, these physical property characteristics of fluorophores are often difficult to manage and predict in the thin solid film owing to their strong tendency to aggregate among π-conjugated planar aromatic moieties that cause either the concentration- or self-quenching effect of excited states or luminescence in the condensed phase. Only certain highly bulky and geometrically hindered π-conjugated chromophore examples possessing restricted or distorted intramolecular rotation bonding units with steric hindrance were found to be fluorescence-active in different physical forms. This led to the retention or increase of fluorescence emission instead of suffering the high concentration-quenching effect upon either one-photon [14] or two-photon [15,16] excitation in the aggregated state as compared with that in the solution. Accordingly, a significant structural modification of organic fluorophores is necessary in order to generate appreciable solid-state nonlinear optical absorptivity, photoluminescence (PL), and electroluminescence (EL) and improve the corresponding quantum yields.

**Scheme 1:**
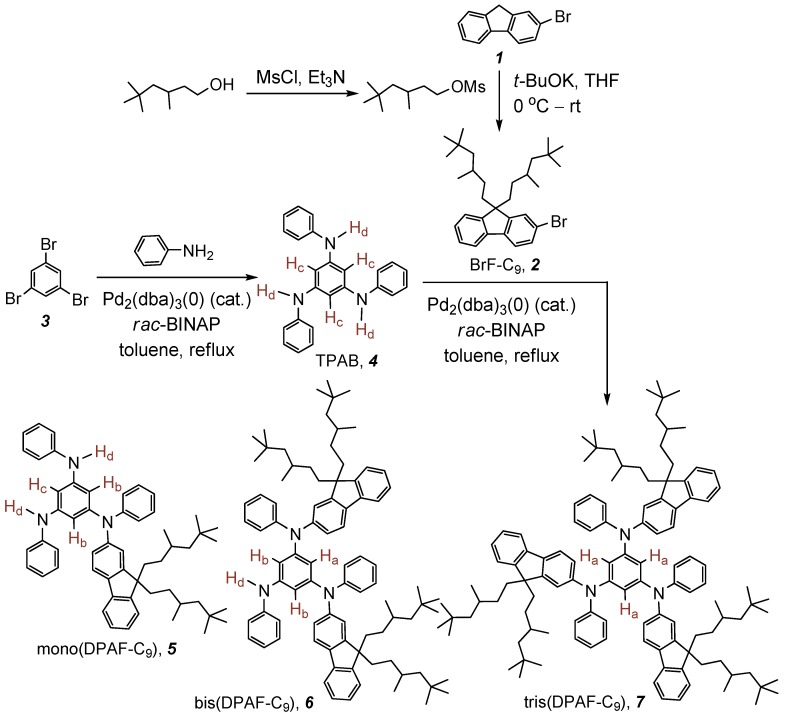
Synthetic route of tris(DPAF-C_9_) **7**.

Structurally branched and dendrimeric design of molecular fluorophores was demonstrated to achieve a large value of simultaneous two-photon absorption (2PA) cross sections during photoexcitation in the NIR region that led to the release of up-converted fluorescence in visible wavelengths. These photophysical properties were associated with the unique nonlinear optical and 2γ-photodynamic sensitizing features. A number of structural variation were demonstrated, including a molecular approach in a multibranched [17,18], octapolar [19,20,21,22], dendrimeric [23,24,25,26], or spiro [27] pattern. Branched and π-conjugated fluorophores with semiconducting charge-transport, light-emission, and high glass-transition temperature characteristics were also of high interest as novel host materials for light-emitting diodes [28]. They consist of a luminescent chromophore at the core region with highly branched conjugated dendron branching side-groups that gave enhanced effects of light emission and charge transport [29]. Using the similar approach, we proposed a molecular configuration with geometrically branched chromophores using a *C*_3_ symmetrical 1,3,5-triaminobenzene ring as the center core for the connection of three fused 2-diphenylaminofluorene moieties, leading to a new class of tris(DPAF-C_9_) **7** structure (Scheme 1). The branched donor fluorophore **7** having high torsional stress at the central core region forces all fluorene chromophore groups located at the outer-edge area to a non-coplanar 3D-configuration. This should minimize the direct contact or π-stacking of the fluorene ring with each other during molecular aggregation and prevent the excimer formation while allows intermolecular interactions of photoluminescent fluorene moieties. We expect well-defined intramolecular and intermolecular space-separation of all diphenylaminofluorene moieties from each other, owing to the pre-designed geometrical configuration around the central benzene ring, should effectively increase the light-exposure surface area of the material and minimize the self-quenching effect of its photoexcited state in a high-concentration form or solids.

## 2. Results and Discussion

Photophysical properties associated with highly enhanced NLO absorption cross sections in the NIR region were achieved by the attachment of one or multiple 9,9'-dialkyl-2-diphenylaminofluorene moieties on a spherical C_60_ molecule using a periconjugated keto-bridging unit [30]. It also indicated the improvement of light harvesting efficiency by using hindered and branched 3',5',5'-trimethylhexyl (C_9_) arms to maintain the space-separation of fluorophores intramolecularly within the nanostructure and intermolecularly during possible cluster formation or aggregation in solution. These conclusions became the basis of our new molecular design of tris(DPAF-C_9_) analogous structures **5**–**7**. In this case, the central benzene ring was shared by two and three phenylaminofluorene antenna unit in the molecular structure of **6** and **7**, respectively, as a fused dendron. Since tris(DPAF-C_9_) **7** is highly fluorescence emissive, it can be utilized as a charge-injecting host fluorophore for the study of PLED devices.

The synthetic route for the preparation of asymmetrical chromophore mono(DPAF-C_9_) **5** and bis(DPAF-C_9_) **6**, and *C*_3_-symmetrical chromophore tris(DPAF-C_9_) **7**, having both dialkylated fluorenyl and phenyl groups on each nitrogen atom of a central 1,3,5-triaminobenzene core was performed, as shown in Scheme 1. Alkylation of 2-bromofluorene **1** with mesylated 3,5,5-trimethylhexanol giving 2-bromo-9,9-bis(3',5',5'-trimethylhexyl)fluorene, BrF-C_9_ **2**, was carried out using potassium *t*-butoxide as a base. The reaction proceeded in a high yield of 90%. The synthesis of 1,3,5-tris(phenylamino)benzene, TPAB **4**, was reported previously [31,32]. However, in order to improve the yield of product, we employed an modified procedure of the palladium-catalyzed amination reaction with tris(dibenzylideneacetone)dipalladium(0) [Pd_2_(dba)_3_(0)] as the catalyst. The reaction was carried out by using five equivalents (excess) of aniline in respect to the quantity of 1,3,5-tribromobenzene **3** in the presence of *rac*-2,2'-bis(diphenylphosphino)-1,1'-binaphthyl (*rac*-BINAP) that resulted in a good tendency for the tris-product formation. Accordingly, a higher yield of **4** in 75% was obtained. A similar palladium-catalyzed amination was also applied in the next step synthesis. For instance, the reaction of TPAB with six equivalents of BrF-C_9_ in dry toluene at refluxing temperature for a period of 72 h was conducted to afford the final product tris(DPAF-C_9_) **7**. The crude product of **7** was then purified subsequently by column chromatography (silica gel) using only hexane and followed by hexane–ethyl acetate (9:1) as the eluent to give light yellow solids or clear thick sticky gel-like paste, while residual solvents are present, in a yield of 88%.

For the synthesis of asymmetrical fluorophores, a different equivalent ratio of starting materials TPAB and BrF-C_9_ in 1:2 was applied for the preparation of bis(DPAF-C_9_) **6** and in 1:1 for the synthesis of mono(DPAF-C_9_) **5**. In the case of the compound **6**, similar reaction conditions as those for **7** in dry toluene were performed to give the product bis(DPAF-C_9_). The crude thick liquid of **6** was purified by column chromatography (SiO_2_) using hexane–ethyl acetate (9:1) as the eluent to yield white to light yellow glassy solids in 52%. For the synthesis of mono(DPAF-C_9_) **5**, a shorter solvent refluxing period of 48 h was used for the reaction of TPAB with one equivalent of **2** in dry toluene, giving the product of mono(DPAF-C_9_) **5** as white to light yellow glassy solids in a yield of 68% after column chromatography (SiO_2_) purification using hexane–ethyl acetate (9:1) initially and then 7:3 as the eluent. All products **5**, **6**, and **7** were found to be photosensitive in the presence of electrophilic solvents and stable as a dry solid. Therefore, dark conditions were necessary during the workup and purification procedures.

Structural characterization of **5**, **6**, and **7** was made using various spectroscopic techniques. By comparison between the IR spectra of tris(DPAF-C_9_) **7** and TPAB, the introduction of dialkylated fluorenyl moiety to **4** was clearly confirmed by the appearance of strong aliphatic C–H stretching vibration bands centered at 2953 cm^−1^ (supporting information) that was absent for TPAB. It also accompanied with several characteristic bands at 3063 and 1583 cm^−1^ assigned, for aromatic C-H stretching and C=C absorption, respectively. Anti-symmetric deformations of CH_3_ groups and scissor vibrations of CH_2_ groups appeared as medium intensity bands centered around 1493 (s) and 1450 cm^−1^, while symmetric deformations of CH_3_ groups exhibited the absorption around 1363 (m) cm^−1^. Two other bands at 1294 (m) and 1249 (m) were assigned to the asymmetric stretching vibrations of C-N-C moieties. Close resemblance of these IR absorption bands was observed for both mono(DPAF-C_9_) **5** and bis(DPAF-C_9_) **6** indicating high similarity of functional groups consisting of DPAF-C_9_ and TPAB moieties. All compounds **5**–**7** showed strong C–H out-of-plan deformation bands at 756 and 711 cm^−1^, for example, for **7**. Furthermore, systematic decrease in intensity of the broad N–H absorption band centered at 3403 and 3375 cm^−1^ for TPAB to 3396 cm^−1^ for **5**, 3402 cm^−1^ for **6**, and complete disappearance for 7, indicated clearly the corresponding progressive amination of fluorenyl moieties (F-C_9_) from **5** to **7**.

**Figure 1:**
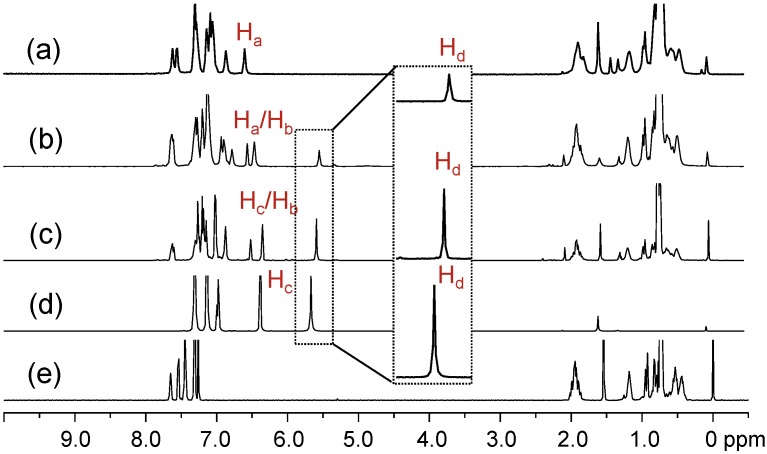
^1^H-NMR spectra of (a) tris(DPAF-C_9_) **7**, (b) bis(DPAF-C_9_) **6**, (c) mono(DPAF-C_9_) **5**, (d) TPAB **4**, and (e) BrF-C_9_ **2**.

^1^H-NMR spectra of the precursor molecules TPAB and BrF-C_9_ were used as the reference, as shown in Figure 1d,e, for the comparison with those of mono-, bis-, and tris(DPAF-C_9_). The most critical evidence of the sequential attachment of 9,9-bis(3',5',5'-trimethylhexyl)fluorene dendrons to **4** was given by the systematic decrease in the integrated intensity ratio of the singlet signal at δ 5.67, assigned for the chemical shift of N–H (H_d_) protons of TPAB (Figure 1d). The peak intensity of this peak matched well with the proton ratio of H_d_ as two, one, and zero for **5** (at δ 5.56, Figure 1c), **6** (at δ 5.52, Figure 1b), and **7** (Figure 1a), respectively. The spectra also indicated the corresponding ratio increase of C_9_-alkyl proton peaks at δ 0.4–2.1 in intensity going from **5** to **7** with roughly similar multiplet peak patterns as those of the parent BrF-C_9_. Characteristics of the central benzene protons can be used for the analysis of the relative geometrically configuration of fluorenyl rings with respect to each other. In the case of mono(DPAF-C_9_) **5** (Figure 1c), two peaks centered at δ 6.33 and 6.50 in a peak integration ratio of 2:1 were clearly detected that allowed us to assign them as the chemical shifts of H_b_ and H_c_, respectively, with the latter having a slight down-fielded shift of 0.18 ppm from that of the singlet H_c_ of **4** at δ 6.32. As the number of fluorene addend (F-C_9_) increased to the structure of bis(DPAF-C_9_) **6**, the central benzene protons changed to H_a_ (fluorene rings at both sides, 1H) and H_b_ (one fluorene ring and one phenyl ring at either sides, 2H) types. Two peak groups located at δ 6.42 (broad singlet) and 6.53 (singlet) in an integration ratio of 2:1 (Figure 1b) allowed a clear assignment to the chemical shift of H_b_ and H_a_, respectively. The peak broadening and splitting at δ 6.42 revealed two non-equivalent H_b_s, perhaps, as the result of a mixture of two possible geometrical conformational isomers of **6** with two fluorene rings either at a *cis*-conformation (up-up) or *trans*-conformation (up-down) since free rotation around the benzene–N bond is unlikely to occur. In addition, an increased complexity and number of fluorenyl proton peaks at δ 6.70–7.70 also supported the existence of two non-equivalent geometrical conformers. Interestingly, the only major product of tris(DPAF-C_9_) **7** isolated showed full disappearance of N–H_d_ proton signal at δ 5.5–6.0 and a singlet H_a_ proton peak at δ 6.55 (Figure 1a) in a similar chemical shift as that of H_a_ in **6**. The former indicated the completion of palladium-catalyzed amination reactions with three equivalents of light-harvesting DPAF-antenna moieties. The latter revealed three equivalent H_a_s that led us to suggest a *cis*-conformation (geometrically at the same side of the central benzene ring as up-up-up configuration for three fluorenyl rings) for the structure of **7** (more discussion below by structural simulation and calculation). Even though the peak and coupling pattern were more complex in the aromatic region of δ 7.0–8.0 in the spectrum as compared to those of the precursor TPAB, however, without a symmetrical environment for three fluorenyl rings in the structure of **7**, the number of aromatic proton peaks each with coupling multiplet should lead to a much more complicated spectrum than that of **6** in the same chemical shift region. Therefore, the observation of only a limit number of fluorenyl aryl proton peak groups at δ 6.82–7.57 with an overall higher proton integration ratio to the peak of H_a_ supported the argument of a quasi-symmetrical *cis*-conformation for **7**.

**Figure 2:**
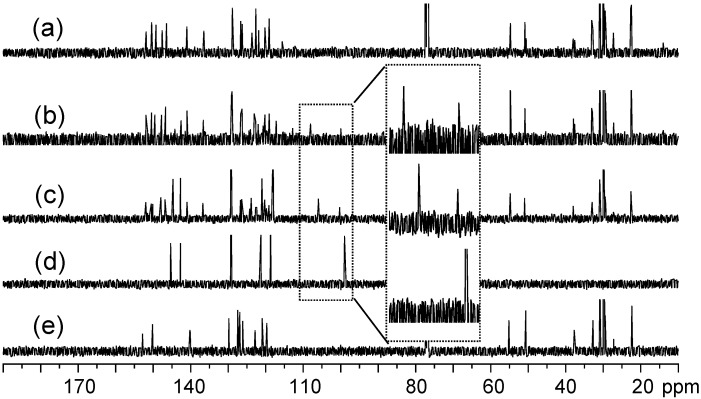
^13^C-NMR spectra of (a) tris(DPAF-C_9_) **7**, (b) bis(DPAF-C_9_) **6**, (c) mono(DPAF-C_9_) **5**, (d) TPAB **4**, and (e) BrF-C_9_ **2**.

**Figure 3:**
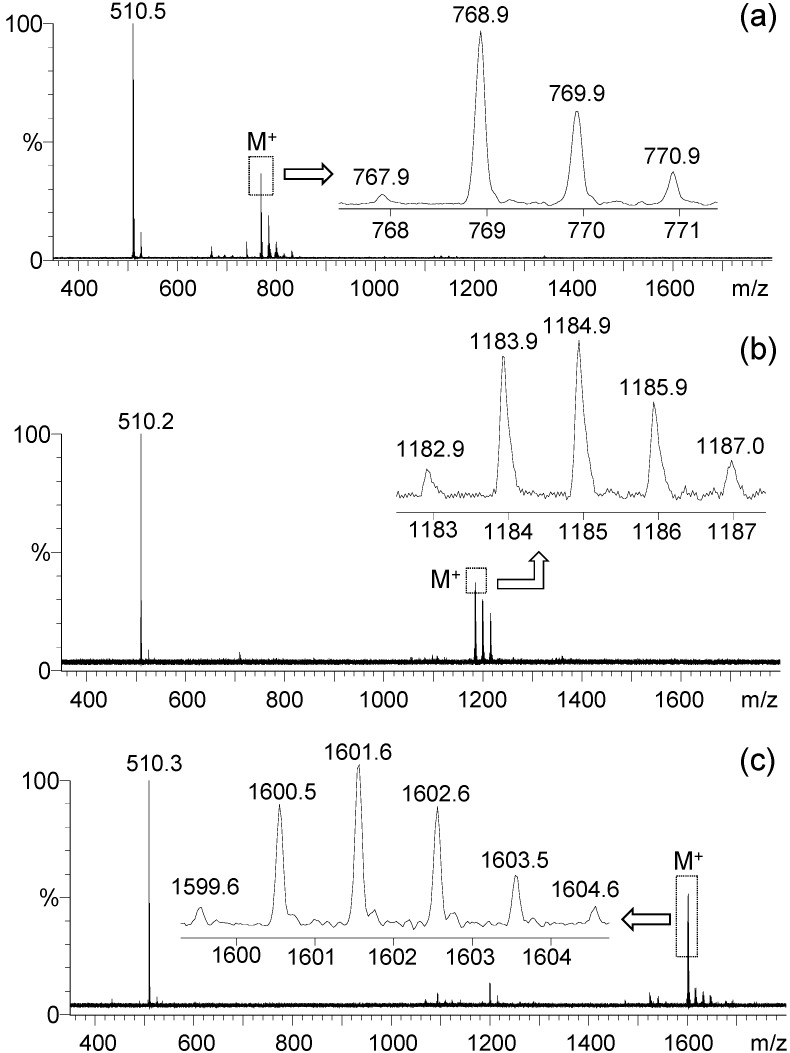
MALDI–TOF mass spectra of (**a**) mono(DPAF-C_9_) **5**, (**b**) bis(DPAF-C_9_) **6**, and (**c**) tris(DPAF-C_9_) **7**.

^13^C-NMR spectra of the precursor molecules TPAB were also used as the reference for the comparison with those of mono-, bis-, and tris(DPAF-C_9_). Attachment of one fluorenyl ring to TPAB led clearly to two sets of aromatic carbon (sp^2^) peaks at δ 115–155 in the ^13^C-NMR spectrum of **5** (Figure 2c). Chemical shifts of the group of five sp^2^ carbon peaks having higher peak intensity were roughly identical to those of TPAB (Figure 2d). This allowed us to assign the rest of multiple peaks in lower peak intensity to the fluorenyl carbons. The relative intensity of these two peak groups was found to increase progressively for the latter as the number of attached fluorenyl ring increased from **5**, **6** (Figure 2b), to **7** (Figure 2a), consistent with their corresponding structural composition. Upon the attachment of one DPAF-C_9_ to **4**, the central phenyl carbon (C_c_) peak of TPAB at δ 99.0 was found to split and shift down-field to δ 105.9 (C_b_) and 100.3 (C_c_), in an intensity ratio of roughly 2:1 consistent with the structure of **5**, in Figure 2c of mono(DPAF-C_9_). Similar shifts to δ 108.0 (C_a_, Figure 2b) and 100.0 (C_b_) for bis(DPAF-C_9_) **6** and to δ 115.6 (H_a_, Figure 2a) for tris(DPAF-C_9_) **7** were detected. These spectroscopic data provided an additional verification of the structures **5**–**7**. High stability of **5**–**7** was observed during positive ion matrix-assisted laser desorption ionization (MALDI–TOF) mass spectroscopic measurements using 3,5-dimethoxy-4-hydroxycinnamic acid (sinapic acid) as the matrix, as shown in Figure 3a–c, respectively. All spectra of **5**, **6**, and **7** displayed a group of molecular ion mass (MH^+^) peaks at *m/z* 768.9, 1184.9, and 1601.6 in good agreement with the calculated mass of M^+^ as *m/z* 767.52, 1183.86, and 1600.21, respectively. It also showed a clean fragmentation pattern consistent with a main fragmented ion mass peak at *m/z* 510.2–510.5, corresponding to the mass of PhN^+^H-fluorene-C_9_. This revealed the central benzene-N bond as the weakest one to undergo dissociation. The data also provided unambiguous evidence of the successful synthesis of mono-, bis-, and tris(DPAF-C_9_).

Effect of TPAB ratio with respect to BrF-C_9_ on the synthesis of tris(DPAF-C_9_) was also investigated. Our initial synthesis of tris(DPAF-C_9_) was carried out by using an exact three-equivalent amount of BrF-C_9_ in respect to the central TPAB core. It was found that this ratio of starting materials did not lead to a clean product, instead resulted in two different product fractions identified as bis- and tris(DPAF-C_9_) showing H_a_ and H_b_ proton peaks (supporting information) at δ 6.56 and 6.48, respectively, slightly down-field shifted in the mixture from those of the corresponding pure sample. By the integration ratio of these two peaks, the major product **7** and the minor product **6** were accounted for 69.0% and 31.0%, respectively. Accordingly, we undertook the TPAB ratio-dependent evaluation of the production formation with three additional ratio of 1:2, 1:4, and 1:6 (excess). As a result, the decrease of TPAB quantity applied to 1:2, the H_b_ peak intensity at δ 6.48 increased significantly indicating the presence of mono(DPAF-C_9_) in addition to bis(DPAF-C_9_) as a mixture. Contrarily, the H_b_ peak decreased sharply in intensity, corresponding to the yield of **6** as 17.3%, as the TPAB ratio being increased to 1:4. We found that a full reaction conversion to **7** became possible by using a TPAB ratio of 1:6, leading to the complete disappearance of H_b_ peak in the product spectrum.

**Figure 4:**
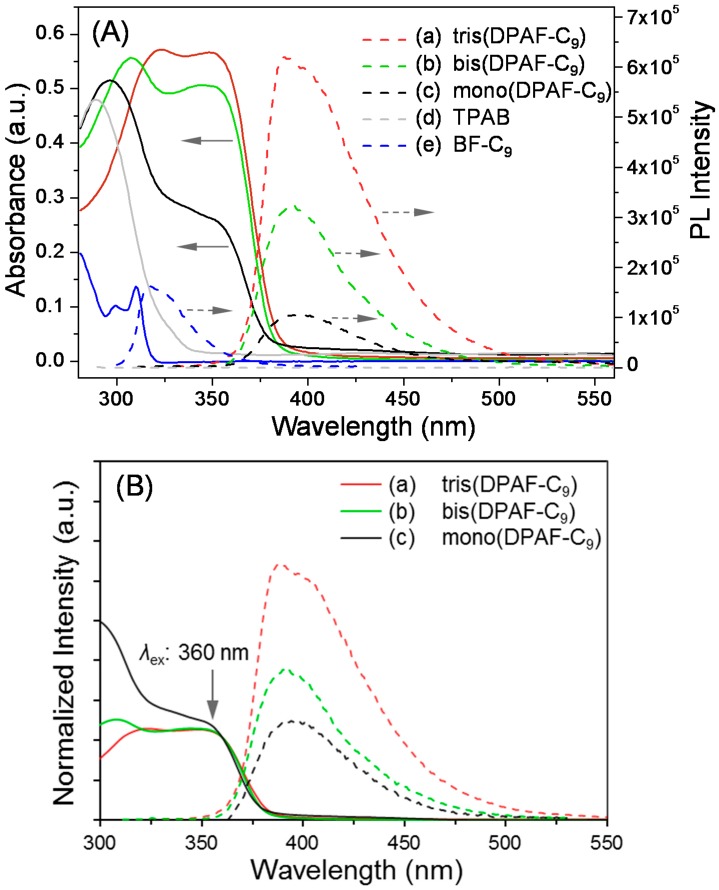
(**A**) UV-vis spectra (solid lines) and photoluminescence (dotted lines) of (a) tris(DPAF-C_9_) **7**, (b) bis(DPAF-C_9_) **6**, (c) mono(DPAF-C_9_) **5**, (d) TPAB **4**, and (e) BrF-C_9_ **2** in CHCl_3_ at a concentration of 1.0 × 10^−5^ M and (**B**) normalized absorption and PL intensity of **5**–**7** at the excitation wavelength of 360 nm.

UV-vis spectra of TPAB **4**, mono-**5**, and bis-**6**, and tris-**7** collected at a concentration of 1.0 × 10^‒5^ M in CHCl_3_ were shown in Figure 4A (solid lines). Chemical modification of TPAB with one or more fluorenyl moieties converted the secondary to corresponding tertiary amines that led to a gradual bathochromic shift from the absorption of **4** centered at 289 nm (λ_max_, ε = 4.80 × 10^4^ L·mol^−1^·cm^−1^) to that of **5** at 297 nm (ε = 5.16 × 10^4^), of **6** at 308 nm (ε = 5.56 × 10^4^), and of **7** at 323 nm (ε = 5.71 × 10^4^ L·mol^−1^·cm^−1^). It also accompanied with a n ew band centered at 349 nm (ε = 2.64 × 10^4^) for **5**, 348 nm (ε = 5.07 × 10^4^) for **6**, and 348 nm (ε = 5.66 × 10^4^ L·mol^−1^·cm^−1^) for **7**. Intensity of the latter band was found to increase progressively from **5** to **7** corresponding to the increasing number of fluorenyl moiety that allowed us to assign this peak to the characteristic absorption band of F-C_9_ moiety. With a new electron-releasing 3,5-diaminophenylamino group being bonded at C_2_ position of a F-C_9_, these bands presented a large bathochromic shift from those of BrF-C_9_ (λ_max_ 299 and 310 nm) with an electron-withdrawing bromo group instead at the same carbon position. The shift also corresponds to an extended fluorenyl ring system interconnected by a 1,3,5-triaminobenzene central core, consistent with the structure of **5**–**7** synthesized.

All mono-**5**, and bis-**6**, and tris(DPAF-C_9_) are highly photoluminescent (PL) chromophores each showing an emission band at λ_em,max_ 391.2, 390.8, and 390.1 nm, respectively, (Figure 4A, dashed lines; λ_ex_ = 360 nm). Intensity of the band increased roughly in proportion to the number of F-C_9_ rings substituted to TPAB. For the clarity, the absorption intensity and relative PL intensity of compounds **5**–**7** were normalized at the excitation wavelength of 360 nm as shown in Figure 4B. Partly owing to the molecular rigidity in the particular stereo-configuration with partial orbital overlap via unpaired electrons of amino groups at the center core of **7** as compared with those of **5** and **6**, an obvious enhancement in the PL intensity (Figure 4B-a) was detected. Similar enhancement in a lesser extent was also observed in the bis(DPAF-C_9_) **6** (Figure 4B-b) in relation to that of mono(DPAF-C_9_) **5**. This revealed emission-independency among F-C_9_ units of **7** with no π-stacking related excimer or self-quenching effect, presumably, owing to the geometrical stereo-conformation of F-C_9_ moieties with respect to each other that prevented the close in-phase π-π plane contact of fluorene rings and yielded high PL in significant intensity. Unlike tris(DPAF-C_9_), PL arising from the benzene and phenyl moieties of TPAB was fully quenched (Figure 4A-d at the baseline) providing evidence of strong intermolecular hydrogen bonding interactions between nitrogen and hydrogen among molecules, leading to a strong self-quenching effect. Accordingly, the S_0_ → S_1_ band gaps (0,0) of **5**, **6**, and **7** can be estimated from the crossover onset position of their absorption and emission edges in CHCl_3_ and were found to be 3.24, 3.26 and 3.28 eV, respectively.

The fluorescent emission wavelength of *cis*-tris(DPAF-C_9_) was found in a close range to that of PVK with both HOMO (−4.81 eV) and LUMO energy level (−1.57 eV) in 1.0 and 0.7 eV, respectively, higher than those of PVK. The former is only slightly lower than that of Ir(ppy)_3_ (−4.9 eV). Therefore, this donor chromophore may serve as the secondary blue hole-transporting material, which is capable of minimizing the aggregation-related self-quenching effect, in the modulation of PLED performance (supporting information Figure S5).

**Figure 5:**
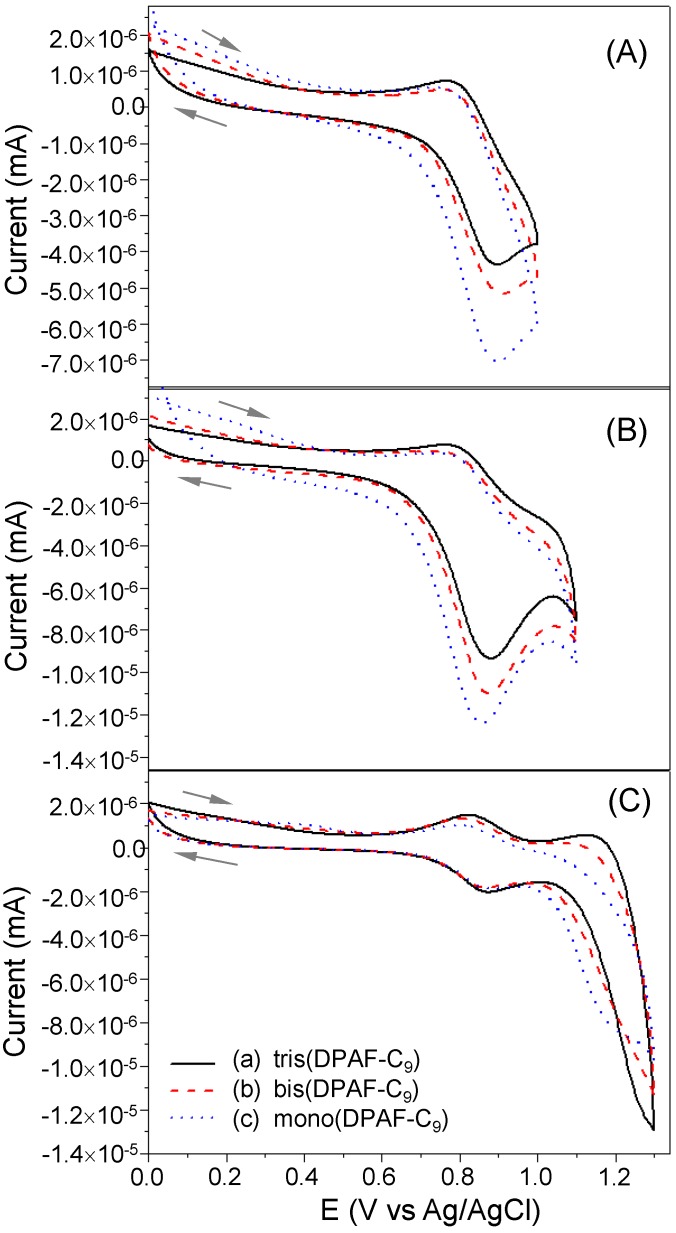
Cyclic voltammograms (CV) of (**A**–**C**) at different voltages vs Ag/AgCl with (a) tris(DPAF-C_9_) **7**, (b) bis(DPAF-C_9_) **6**, and (c) mono(DPAF-C_9_) **5** solution in a concentration of 1.75 × 10^‒3^ M in CH_3_CN–CH_2_Cl_2_, containing (*n*-butyl)_4_N^+^-PF_6_^‒^ electrolyte (0.4 M), using Pt as working and counter electrodes and Ag/AgCl as the reference electrode at a scan rate of 10 mV/s.

We also carried out the cyclic voltammetry (CV) measurement of mono-**5**, bis-**6**, and tris-**7** in a solvent mixture of CH_2_Cl_2_–CH_3_CN in the presence of (*n*-butyl)_4_N^+^-PF_6_^‒^ electrolyte, using Pt as working and counter electrodes and Ag/AgCl as the reference electrode. The CV characteristics of compounds **5**–**7** under variation of the cyclic oxidation voltage vs Ag/AgCl from 1.0 to 1.3 V were evaluated, as shown in Figure 5A–C. As a result, only one consistent reversible redox wave was detectable in a similar current range (0.0–1.1 V) for all three compounds indicating that only one F-C_9_ ring moiety was participating the oxidation–reduction cycle at the same concentration regardless its structure being mono-, bis-, or tris-adduct of TPAB. This revealed full electrochemical independency of all F-C_9_ rings in the structure. By taking the most stable (symmetrical) and reversible first cyclic wave of Figure 5C as an example, a nearly identical onset oxidation potential (*E*_ox_^onset^) was measured for **5**, **6**, and **7** as 0.83 V. Under the same CV conditions, the first half-wave redox potential of ferrocence reference sample was measured as 0.82 V. Therefore, by taking the oxidation potential value of ferrocence at −4.8 eV below the vacuum level as the reference, the HOMO levels of mono-**5**, bis-**6**, and tris-7 were calculated as a nearly identical value of −4.81 eV that was summarized in Table 1. Furthermore, the optical energy gap (*E*_opt_, S_0_ → S_1_ band gap) can be obtained and calculated from the absorption onset of all compounds **5**, **6**, and **7**, via Figure 4, in a value of 3.28, 3.26, and 3.24 eV, respectively, as discussed above. Consequently, corresponding LUMO levels were then calculated as −1.53, −1.55, and −1.57 eV, respectively.

**Table 1:** Electrochemical properties of mono-**5**, bis-**6**, and tris(DPAF-C_9_) **7**.

Compound	*E*_ox_^onset^ (V)	HOMO (eV) *^a^*	*E*_opt_ (eV) *^b^*	LUMO (eV)
Tris(DPAF-C_9_) **7**	0.83	*−*4.81	3.24	*−*1.57
Bis(DPAF-C_9_) **6**	0.83	*−*4.81	3.26	*−*1.55
Mono(DPAF-C_9_) **5**	0.83	*−*4.81	3.28	*−*1.53

*^a^* Estimated from the onset oxidation potential; *^b^* Estimated from the onset of absorption edge.

**Figure 6:**
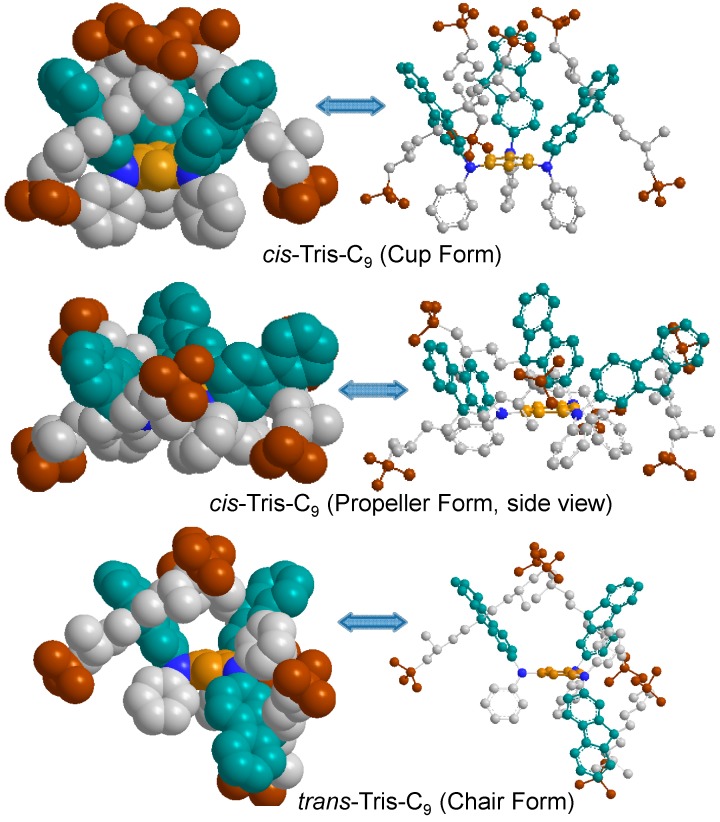
Optimized geometrical structures of tris(DPAF-C_9_) **7**, by calculation, showing a different degree of interaction force among terminal *t*-butyl groups (brown).

In the consideration of the structural configuration, it is reasonable to expect two possible nonplanar geometrical stereoisomers as, namely, tris-*trans*- (with two fluorenyl rings at the same side of the central benzene ring and one fluorenyl rings located at the opposite side as an up-up-down configuration) and tris-*cis*-conformation (geometrically at the same side of the central benzene ring as an up-up-up configuration for all three fluorenyl rings) analogues, for the structure of tris(DPAF-C_9_) **7** in spite of only one chromatographic spot being detected on TLC (silica gel, toluene–hexane, 5:95). This observation clearly suggests that the 3D-structure of **7** may not be a planar configuration with the fluorenyl and/or the phenyl rings being possibly twisted relative to the center core position, forming a pair of *cis/trans* stereo-conformers or isolable isomers, as discussed in Figure 6. In order to estimate the C-C bond rotational barrier energy of tris(DPAF-C_9_) between possible stereo-conformers, we conducted the variable temperature NMR measurement in the range of 300–335 K in CDCl_3_. However, we did not observe any clear difference except small peak broadening. This result suggested that the rotational barrier energy is high enough to restrict the *cis*–*trans* isomerization and allowed the isolation of a single isomer at least in room temperature.

As we described above, uncongested ^1^H-NMR spectrum of tris(DPAF-C_9_) **7** revealed a *C*_3_-symmetrical *cis*-conformation for its structure. Our puzzle existed regarding to the exclusive formation of, supposedly, sterically unfavorable *cis*-isomer. Accordingly, we undertook the comparison of formation energy by calculation on the 3D-structure of three plausible isomers of tris(DPAF-C_9_) **7**, as shown in Figure 6. Two *cis*-forms, especially a *cis*-(*cup*-)form, seem to be more crowded than *trans*-(*chair*-)form. Surprisingly, heat of formation (Δ*H*_f_) calculated by the semi-empirical PM3 method revealed unexpectedly high stability of the *cup*-form with other forms in an order of *chair* ≈ *cup* > *propeller* for C_9_ substituents (−9.19, −9.13, and −4.40 kcal/mol, respectively) and the highest stability of *cup*-forms (*cup* > *chair* > *propeller*) for the slightly less crowded *n*-C_6_, C_7_, and C_8_ substituents. Such *cup*-form formation tends to be more preferable as the alkyl chains at 9-fluorenyl position become longer as can be seen in the differential heat of formation ΔΔ*H*_f_ for both *cup*–*propeller* and *cup*–*chair* energy gaps (Table 2). These results imply that hydrophobic interaction between three alkyl chains as well as CH/π interaction between the alkyl group and the fluorenyl ring plays an important role for the preferential formation of the *cup-*form. The DFT calculation at B3LYP/6-31G* level of theory for some alkyl groups resulted in the similar tendency, although the values suggested the slightly more stable *chair*-form even for *n*-C_6_ group as compared with the *cup*-form, probably due to the lack of consideration on the solvent polarity which affects to hydrophobic interactions (Table 3).

**Table 2:** Calculated heat of formation for tris(DPAF-C_n_) *^a^*.

R *^b^*	Δ*H*_f_ (kcal/mol)			ΔΔ*H*_f_ (kcal/mol)	
(C_n_)	*cis*		*trans*	*cup*–*propeller*	*cup*–*chair*
	*cup*	*propeller*	*chair*		(*cis*–*trans*)
Me	258.38	253.67	247.85	4.71	10.53
Et	231.59	223.91	233.48	7.68	1.89
*n*-C_6_	87.01	95.12	93.67	−8.11	−6.66
C_7_	59.59	64.00	60.07	−4.41	−0.48
C_8_	22.23	28.10	22.52	−5.87	−0.29
C_9_	−9.13	−4.40	−9.19	−4.73	0.06

*^a^* Calculated by semi-empirical PM3 method using Spartan 08. *^b^* Substituents at 9-fluorenyl position. C_7_: 3-methylhexyl group (3-Me); C_8_: 5,5-dimethylhexyl group (4-*t*-Bu); C_9_: 3,5,5-trimethylhexyl group (3-Me-4-*t-*Bu).

**Table 3:** DFT calculation of heat of formation for tris(DPAF-C_n_) *^a^*.

R *^b^*	Δ*H*_f_ (kcal/mol)		ΔΔ*H*_f_ (kcal/mol)
(C_n_)	*cis* (*cup*)	*trans* (*chair*)	*cup*–*chair*
Et	−1922585.7	−1922609.1	23.4
MeOC_2_H_4_	−2353750.4	−2353762.0	11.6
*n*-C_6_	−2514673.7	−2514677.3	3.6
C_9_	−2958697.2	−2958703.8	6.6

*^a^* Calculated at B3LYP/6-31G* level of theory using SPARTAN08. *^b^* Substituents at 9-fluorenyl position.

**Figure 7:**
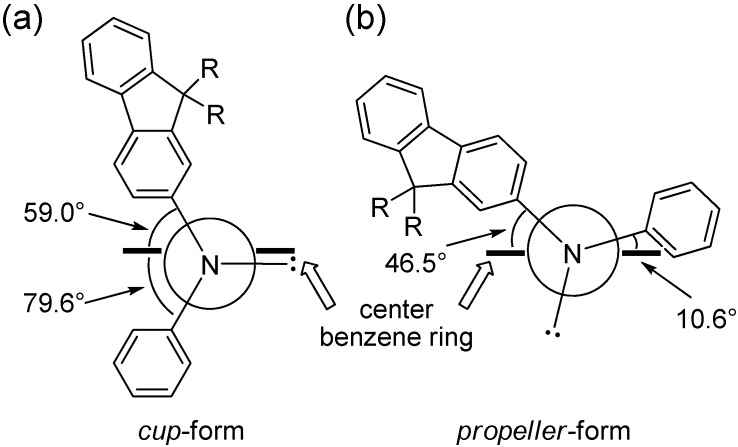
Torsional (dihedral) angles between aryl–nitrogen bond and center benzene ring for (**a**) *cup*-form and (**b**) *propeller*-form of tris(DPAF-C_9_) **7**.

The unexpected stability of *cup*-form may be explained by the torsional angle of the center benzene ring defined by the position of aryl groups on nitrogen atoms. The calculated torsional (dihedral) angles between the aryl–nitrogen C–N bond and the center benzene ring C–C bond for two *cis*-conformers of tris(DPAF-C_9_) (*cup*-form and *propeller*-form) obtained from the optimized 3D-structure are illustrated in Figure 7. The angle strain of the *cup*-form was found to be much less than that of the *propeller*-form. The angle strain of *trans*-conformers is somewhat higher than that of the symmetrical *cup*-form due to unavoidable unsymmetrical conformation of the *chair*-form. Therefore, the highly symmetrical *cis*-*cup*-isomer of tris(DPAF-C_9_) can be formed exclusively.

In summary, above explanations for the preferential *cis*-*cup*-formation are supported by results of the calculated heat of formation and dihedral angle, which are based on the thermodynamically controlled mechanism. However, the restricted bond rotation may not give the thermodynamically stable isomer under the present condition. Therefore, an alternative explanation for the preferential *cis*-*cup*-formation of tris(DPAF-C_9_) is also considered based on the kinetically controlled mechanism in the critical amination reaction. Fluorenyl substitution at three phenylamino groups on the central benzene ring of TPAB occurs one by one to form mono(DPAF-C_9_), bis(DPAF-C_9_), and tris(DPAF-C_9_) sequentially. Owing to the unrestricted rotation of phenylamino group, two phenyl groups on nitrogen atoms can be escaped from a larger fluorenyl substituent group via the steric hindrance. Consequently, the tendency of subsequent substitution of a second fluorenyl group should favor the formation of *cis*-bis(DPAF-C_9_). This preferred configuration may hinder its isomerization to the *trans*-form due to the restricted bond rotation. Similarly, the third substitution of the last fluorenyl group should lead to the selective product of *cis*-tris(DPAF-C_9_) (supporting information).

## 3. Experimental 

*Materials.* Reagents and solvent of 3,5,5-trimethylhexanol, methanesulfonyl chloride, triethylamine, 2-bromofluorene, 1,3,5-tribromobenzene, *rac*-2,2'-bis(diphenylphosphino)-1,1'-binaphthyl (*rac*-BINAP), tris(dibenzylideneacetone)dipalladium(0) [Pd_2_(dba)_3_(0)], aniline, and dichloroethane were purchased from Aldrich Chemicals (Milwaukee, WI, USA) and used without further purification. All other chemicals were purchased from Acros Ltd. (Fair Lawn, NJ, USA). The anhydrous grade solvent of THF was refluxed over sodium and benzophenone overnight and distilled under reduced pressure (10^−1^ mmHg). 

*Spectroscopic Measurements.* ^1^H-NMR and ^13^C-NMR spectra were recorded on either a Bruker Avance Spectrospin–200 or Bruker AC-300 spectrometer (Bruker, Billerica, MA, USA). UV-vis spectra were recorded on a U-3410 UV spectrometer (Hitachi, Schaumburg, IL, USA). Infrared spectra were recorded as KBr pellets on a Nicolet 750 series FT-IR spectrometer (Thermo Electron Corporation, Madison, WI, USA). Photoluminescence (PL) spectra were measured using a PTI (Birmingham, NJ, USA) Fluorescence Master Systems connected with a photomultiplier (914 Photomultiplier Detection System), with a xenon short arc lamp as the excitation source. Mass spectroscopic measurements were performed by the use of positive ion matrix-assisted laser desorption ionization (MALDI–TOF) technique on a micromass M@LDI-LR mass spectrometer (Bruker, Billerica, MA, USA). The sample blended or dissolved in the matrix material was irradiated by nitrogen UV laser at 337 nm with 10 Hz pulses under high vacuum. Mass ion peaks were identified for the spectrum using the MassLynx v4.0 software. In a typical experiment, the samples of 5, 6, and 7, were dissolved in CHCl_3_ in a concentration of 1.0 mg/mL. The matrix of 3,5-dimethoxy-4-hydroxycinnamic acid (sinapic acid) was dissolved in THF in a concentration of 10 mg/mL. The solution of matrix (1.0 mL) was taken and mixed with the sample solution (0.1 mL) prior to the deposition on a stainless-steel MALDI target probe. It was subsequently dried at ambient temperature.

### Synthetic Procedures

#### Synthesis of 3,5,5-Trimethylhexyl Methanesulfonate

To a stirred solution of 3,5,5-trimethylhexanol (5.0 g, 34.6 mmol) in anhydrous dichloroethane (75 mL) was added triethylamine (3.85 g, 38.12 mmol). This reaction mixture was cooled to 0 °C in ice bath, follwed by dropwise addition of methane sulfonyl chloride (4.36 g, 38.12 mmol) over a period of 15 min under nitrogen. The reaction mixture was stirred for overnight and quenched by the addition of water. Organic layer was washed with water (2 × 50 mL), dil. hydrochloric acid (1 × 50 mL), saturated sodium bicarbonate (1 × 50 mL), and brine solution (1 × 50 mL). The residue was dried over sodium sulphate and concentrated on a rotavapor to afford crude light brown colored liquid. After chromatographic purification, the product 3,5,5-trimethylhexyl methanesulfonate was obtained in a yield of 95%, as a colorless liquid after vacuum distillation at 130–140 °C. ^1^H-NMR (500 MHz, CDCl_3_) δ 4.25 (t, *J* = 6.8 Hz, 2H), 3.01(s, 3H), 1.77 (dq, *J* = 13.8, 6.9, 6.4 Hz, 1H), 1.67 (q, *J* = 8.0, 6.3 Hz, 1H), 1.57 (dq, *J* = 13.3, 6.6 Hz, 1H), 1.24–1.20 (m, 1H), 1.14–1.10 (m, 1H), 0.97 (d, *J* = 6.6 Hz, 3H), 0.90 (s, 8H), and 0.87–0.76 (m, 1H).

#### Synthesis of 2-Bromo-9,9-bis(3',5',5'-trimethyl-1'-hexyl)fluorene (BrF-C_9_, **2**)

To a solution of 2-bromofluorene **1** (5.0 g, 20.4 mmol) in anhydrous tetrahydrofuran (100 mL) was added potassium *t*-butoxide (5.03 g, 44.80 mmol) at 0 °C. The resulting orange brown colored reaction mixture was stirred for 30 min. To this reaction mixture 3,5,5-trimethylhexyl methanesulfonate (9.98 g, 44.8 mmol) in anhydrous tetrahydrofuran (25 mL) was added dropwise over a period of 30 min. It was stirred for overnight and subsequently diluted with water (50 mL). The product was extracted with ethyl acetate (150 mL), washed with water, and dried over anhydrous sodium sulfate (Na_2_SO_4_). The pale yellow crude product was purified by column chromatography using silica gel as the stationary phase and hexane–ethyl acetate (19:1) as the eluent. The purified product 2-bromo-9,9-bis(3',5',5'-trimethyl-1'-hexyl)fluorene **2**, BrF-C_9_, was obtained as colorless liquid in 90% yield (9.1 g). FT-IR (KBr) *υ*_max_ 3063 (m), 3024 (m), 2953 (s), 2868 (s), 2744 (w), 2,715 (w), 1944 (w), 1905 (w), 1875 (w), 1799 (w), 1749 (w), 1,642 (w), 1599 (m), 1570 (m), 1466 (s), 1443 (s), 1406 (m), 1389 (m), 1364 (s), 1292 (w), 1264 (m), 1246 (m), 1217 (m), 1155 (w), 1132 (w), 1062 (m), 1028 (w), 1005 (m), 972 (w), 935 (w), 877 (m), 822 (m), 781 (m), 758 (m), 736 (s), 643 (w), and 572 (m) cm^−1^; UV-vis (CHCl_3_, 1.0 × 10^−5^ M) λ_max_ (ε) 279 (2.04 × 10^4^), 299 (1.03 × 10^4^), and 310 nm(1.37 × 10^4^ L mol^−1^ cm^−1^); ^1^H-NMR (500 MHz, CDCl_3_) δ 7.70–7.62 (m, 1H), 7.54 (d, *J* = 7.9 Hz, 1H), 7.45–7.43 (m, 2H), 7.31(s, 3H), 2.02–1.87 (m, 4H), 1.22–1.13 (br, 2H), 0.96–0.95 (m, 1H), 0.93–0.92 (m, 1H), 0.84–0.77 (m, 3H), 0.73 (t, *J* = 5.3 Hz, 23H), 0.58–0.50 (m, 2H), and 0.47–0.39 (m, 2H); ^13^C-NMR (125 MHz, CDCl_3_) δ 152.9, 150.2, 140.2, 140.1, 129.8, 127.5, 126.9, 126.1, 122.8, 121.0, 120.9, 119.7, 55.2, 50.74, 50.65, 37.82, 37.79, 37.7, 32.81, 32.75, 30.9, 29.9, 29.5, 29.4, 22.5, and 22.4.

#### Synthesis of 1,3,5-Tris(phenylamino)benzene (TPAB, **4**) 

A mixture of aniline (7.4 g, 79.4 mmol), 1,3,5-tribromobenzene **3** (5.0 g, 15.88 mmol), tris(dibenzylideneacetone) dipalladium(0) [Pd_2_(dba)_3_(0), 0.11 g, 0.25 mol %], *rac*-2,2'-bis(diphenylphosphino)-1,1'-binapthyl (BINAP, 0.22 g, 0.75 mol %), and sodium *t*-butoxide (7.63 g, 79.4 mmol) in anhydrous toluene (250 mL) was heated to refluxing temperature under nitrogen for a period of 72 h. The reaction mixture was cooled to room temperature and washed with water (100 mL). The organic layer was dried over anhydrous sodium sulfate (Na_2_SO_4_). After evaporation of the solvent, a crude brown color solid was obtained. Excess aniline was then removed by distillation at 130 °C under reduced pressure of 10^‒1^ mmHg. It was further purified by chromatography (SiO_2_). The resulting light brown product 1,3,5-tris(phenylamino)benzene **4**, TPAB, was obtained in 75% yield (4.18 g). FT-IR (KBr) *υ*_max_ 3403 (m), 3375 (m), 3078 (w), 3019 (w), 2945 (s), 1612 (m), 1591 (vs), 1518 (m), 1496 (s), 1469 (m), 1431(w), 1456 (m), 1409 (m), 1365 (w), 1295 (m), 1269 (w), 1246 (m), 1,171 (m), 1073 (w), 1030 (w), 897 (w), 834 (w), 820 (w), 802 (w), 756 (m), 721 (w), 700 (m), 688 (m), 613 (w), and 558 (w) cm^−1^; UV-vis (CHCl_3_, 1.0 × 10^−5^ M) λ_max_ (ε) 289 nm (4.80 × 10^4^ L mol^−1^ cm^−1^); ^1^H-NMR (500 MHz, CDCl_3_) δ 7.25 (t, *J* = 7.8 Hz, 6H), 7.08 (d, *J* = 7.7 Hz, 6H), 6.92 (t, *J* = 7.3 Hz, 3H), 6.32 (s, 3H), and 5.60 (s, 3H, N–H); ^13^C-NMR (125 MHz, CDCl_3_) δ 145.4, 142.7, 129.3, 121.3, 118.7, and 99.0.

#### Synthesis of *N*^1^,*N*^3^,*N*^5^-Tris(9,9-di(3',5',5'-trimethyl-1'-hexyl)fluoren-2-yl)-1",3",5"-tris(phenylamino)-benzene [tris(DPAF-C_9_), **7**] 

A mixture of 2-bromo-9,9-bis(3',5',5'-trimethyl-1'-hexyl)fluorene (8.39 g, 17.04 mmol, excess), 1,3,5-tris(*N*-phenylamino)benzene (1.0 g, 2.84 mmol), tris(dibenzylideneacetone)dipalladium(0) [Pd_2_(dba)_3_(0), 0.02 g, 0.25 mol %], *rac*-2,2'-bis(diphenylphosphino)-1,1'-binapthyl (BINAP, 0.04 g, 0.75 mol %), and sodium *t*-butoxide (1.63 g, 17.04 mmol) in anhydrous toluene (75 mL) was heated to refluxing temperature under nitrogen for a period of 72 h. The reaction mixture was cooled to room temperature and washed with water (50 mL). The organic layer was dried over anhydrous sodium sulfate. After evaporation of the solvent, it afforded a crude brown colored paste. The paste was subjected to column chromatography purification using silica gel as the stationary phase and hexane–ethylacetate (9:1) as the eluent, to afford *N*^1^,*N*^3^,*N*^5^-tris(9,9-di(3',5',5'-trimethyl-1'-hexyl)fluoren-2-yl)-1",3",5"-tris(phenylamino)-benzene, tris(DPAF-C_9_) **7**, as light yellow solids or clear thick sticky gel-like paste, while residual solvents are present, in 88% yield (3.98 g). FT-IR (KBr) *υ*_max_ 3,063 (w, aromatic C–H stretching), 3,037 (w), 3,019 (w), 2,953 (vs, aliphatic C–H stretching), 2,865 (s), 1,583 (s, C=C), 1500 (s), 1,493 (s, anti-symmetric deformations of CH_3_ groups and scissor vibrations of CH_2_ groups), 1,450, 1392 (w), 1363 (m, symmetric deformations of CH_3_ groups), 1294 (m, asymmetric stretching vibrations of C–N–C), 1249 (w, asymmetric stretching vibrations of C–N–C), 1212 (w), 1178 (w), 1155 (w), 1037 (w), 1006 (w), 933 (w), 878 (w), 826 (w), 756 (w), 738 (s, C–H out-of-plan deformation), 711 (m, C–H out-of-plan deformation), 693 (m), 628 (w), and 510 (w) cm^−1^; UV-vis (CHCl_3_, 1.0 × 10^−5^ M) λ_max_ (ε) 323 (5.71 × 10^4^) and 348 nm (5.66 × 10^4^ L mol^−1^ cm^−1^); PL (CHCl_3_, 1.0 × 10^−5^ M) λ*_em,_*_max_ 390.1 nm; ^1^H-NMR (500 MHz, CDCl_3_) δ 7.57 (s, 3H, br), 7.51 (d, *J* = 7.5 Hz, 3H), 7.32–7.21 (m, 9H), 7.09–7.01 (m, 18H), 6.82 (t, *J* = 7.2 Hz, 3H), 6.55 (s, 3H, br), 1.92–1.67 (m, 12H), 1.11 (s, 6H), and 0.97–0.39 (m, 96H); ^13^C-NMR (125 MHz, CDCl_3_) δ 151.9, 150.4, 149.3, 147.6, 146.5, 141.0, 136.5, 128.9, 126.7, 126.3, 123.7, 122.7, 121.9, 120.2, 119.1, 115.6, 54.7, 50.9, 50.63, 50.57, 38.0, 37.9, 37.6, 37.5, 33.1, 32.8, 30.9, 30.0, 29.5, 29.3, 27.3, 22.7, and 22.5; MALDI–TOF MS calcd for C_117_H_153_N_3_, *m/z* 1600.2; found, *m/z* 1601.6 (MH^+^).

#### Synthesis of *N*^1^,*N*^3^-Bis(9,9-di(3',5',5'-trimethyl-1'-hexyl)fluoren-2-yl)-1",3",5"-tris(phenylamino)-benzene [bis(DPAF-C_9_), **6**]

A mixture of 2-bromo-9,9-bis(3',5',5'-trimethyl-1'-hexyl)fluorene (0.71 g, 1.42 mmol), 1,3,5-tris(phenylamino)benzene (0.25 g, 0.71 mmol), tris(dibenzylideneacetone)dipalladium(0) [Pd_2_(dba)_3_(0), 0.005 g, 0.25 mol %], *rac*-2,2'-bis(diphenylphosphino)-1,1'-binapthyl (BINAP, 0.01 g, 0.75 mol %), and sodium *t*-butoxide (0.70 g, 7.10 mmol) in anhydrous toluene (50 mL) was heated to refluxing temperature under nitrogen for 72 h. The reaction mixture was cooled to room temperature and washed with water (30 mL). The organic layer was dried over anhydrous sodium sulfate. After evaporation of the solvent, it afforded a crude brown colored semi-solid. It was purified by column chromatography using silica gel as the stationary phase and hexane–ethylacetate (9:1) as the eluent to afford *N*^1^,*N*^3^-bis(9,9-di(3',5',5'-trimethyl-1'-hexyl)fluoren-2-yl)-1",3",5"-tris(phenylamino)benzene, bis(DPAF-C_9_) **6**, as white to light yellow glassy solids in 52% yield (0.43 g). FT-IR (KBr) *υ*_max_ 3402 (m), 3062 (w), 3036 (w), 3010 (w), 2953 (s), 2865 (m), 1585 (s), 1492 (s), 1467 (m), 1450 (m), 1,422 (w), 1392 (m), 1363 (w), 1299 (m), 1252 (m), 1213 (w), 1155 (w), 1079 (w), 1033 (w), 824 (w), 738 (m), 711 (w), 693 (m), and 512 (w) cm^−1^; UV-vis (CHCl_3_, 1.0 × 10^−5^ M) λ_max_ (ε) 308 (5.56 × 10^4^) and 348 nm (5.07 × 10^4^ L mol^−1^ cm^−1^); PL (CHCl_3_, 1.0 × 10^−5^ M) λ*_em,_*_max_ 390.8 nm; ^1^H-NMR (500 MHz, CDCl_3_) δ 7.61–7.58 (m, 4H), 7.34–7.21 (m, 7H), 7.16 (t, *J* = 7.2 Hz, 4H), 7.12–7.01 (m, 10H), 6.90 (t, *J* = 6.8 Hz, 1H), 6.86–6.83 (m, 2H), 6.74 (t, *J* = 6.5 Hz, 1H), 6.53 (s, 1H), 6.42 (s, 2H), 5.51 (s, 1H), 1.98–1.71 (m, 8H), 1.21–1.07 (s, 4H, br), 0.97–0.86 (d, 4H), and ,0.83–0.34 (m, 60H); ^13^C-NMR (125 MHz, CDCl_3_) δ 151.9, 150.5, 149.6, 147.8, 146.7, 142.5, 141.0, 136.6, 129.1, 129.0, 126.7, 126.3, 124.1, 123.1, 122.7, 122.0, 120.6, 120.2, 119.1, 117.2, 112.8, 108.10, 108.0, 100.0, 54.8, 50.9, 50.7, 38.0, 37.9, 37.7, 37.6, 33.1, 32.9, 30.9, 29.9, 29.5, 29.30, 29.27, 27.3, 22.6, and 22.5; MALDI–TOF MS calcd for C_86_H_109_N_3_, *m/z* 1,183.9; found, *m/z* 1,184.9 (MH^+^).

#### Synthesis of *N*^1^-Mono(9,9-di(3',5',5'-trimethyl-1'-hexyl)fluoren-2-yl)-1",3",5"-tris(phenylamino)-benzene [mono(DPAF-C_9_), **5**]

A mixture of 2-bromo-9,9-bis(3',5',5'-trimethyl-1'-hexyl)fluorene (0.36 g, 0.71 mmol), 1,3,5-tris(phenylamino)benzene (0.25 g, 0.71 mmol), tris(dibenzylideneacetone)dipalladium(0) (Pd_2_(dba)_3_(0), 0.005 g, 0.25 mol %), *rac*-2,2'-bis(diphenylphosphino)-1,1'-binapthyl (BINAP, 0.01 g, 0.75 mol %), and sodium *t*-butoxide (0.70 g, 7.10 mmol ) in anhydrous toluene (50 mL) was heated to refluxing temperature under nitrogen for a period of 48 h. The reaction mixture was cooled to room temperature and washed with water (30 mL). The organic layer was dried over anhydrous sodium sulfate. After evaporation of the solvent, it afforded a crude brown semi-solid. It was purified by column chromatography using silica gel as the stationary phase and hexane–ethylacetate (9:1) as the eluent then slowly increased ethyl acetate contents up to 30% to give *N*^1^-mono(9,9-di(3',5',5'-trimethyl-1'-hexyl)fluoren-2-yl)-1",3",5"-tris(phenylamino)benzene, mono(DPAF-C_9_) **5**, as white to light yellow glassy solids in 68% yield (0.37 g). FT-IR (KBr) *υ*_max_ 3396 (m), 3037 (w), 2952 (s), 2865(w), 1585 (vs), 1518 (w), 1495 (s), 1467 (m), 1450 (m), 1421 (w), 1393 (w), 1363 (w), 1301 (m), 1249 (m), 1171 (w), 1078 (w), 1031 (w), 823 (w), 739 (m), 692 (m), 595(w), and 514 (w) cm^−1^; UV-vis (CHCl_3_, 1.0 × 10^−5^ M) λ_max_ (ε) 297 (5.16 × 10^4^) and 349 nm (2.64 × 10^4^ L mol^−1^ cm^−1^); PL (CHCl_3_, 1.0 × 10^−5^ M) λ*_em,_*_max_ 391.2 nm; ^1^H-NMR (500 MHz, CDCl_3_) δ 7.62–7.58 (m, 2H), 7.33–7.11 (m, 14H), 7.00 (d, *J* = 7.5 Hz, 4H), 6.85 (t, *J* = 7.3 Hz, 2H), 6.50 (s, 1H), 6.33 (d, *J* = 1.9 Hz, 2H), 5.56 (s, 2H), 1.98–1.73 (m, 4H), 1.15 (s, 2H), 0.91 (d, *J* = 13.8 Hz, 2H), and 0.84–0.39 (m, 30H); ^13^C-NMR (125 MHz, CDCl_3_) δ 152.0, 150.5, 150.1, 148.0, 146.9, 144.7, 142.7, 141.0, 136.7, 129.3, 129.1, 126.7, 126.4, 124.3, 123.9, 122.7, 122.4, 121.0, 120.3, 119.8, 119.2, 118.0, 105.9, 100.3, 54.8, 51.0, 50.7, 38.0, 37.8, 37.7, 33.2, 33.0, 30.9, 30.0, 29.5, 29.4, 22.6, and 22.5; MALDI–TOF MS calcd for C_117_H_153_N_3_, *m/z* 767.5; found, *m/z* 768.9 (MH^+^).

## 4. Conclusions

A novel highly luminescent tris-fluorenyl ring-interconnected chromophore tris(DPAF-C_9_) **7** was designed and synthesized using a *C*_3_ symmetrical triaminobenzene core as the synthon. The structural moiety bears a close resemblance to that of 2-diphenylamino-9,9-dialkylfluorenyl attachment in the starburst nonlinear photonic materials C_60_(>DPAF-C_n_)_x_, reported previously [33,34,35,36]. The synthetic chromophore **7** was chromatographically purified and characterized as a major stereoisomer possessing a 3D structural configuration of the *cis*-*cup*-conformer showing symmetrical phenyl protons of the central benzene ring. A molecular calculated structure of **7** at B3LYP/6-31G* level of theory based on the optimized 3D structure indicated the unexpected stability of the *cup*-form as explained by the dihedral torsional angle of the central benzene ring that revealed much less angle strain than that of the stereoisomer in a *propeller*-form. Angle strain of the *trans*-*chair*-conformer was found to be higher by the same calculation due to the unavoidable unsymmetrical conformation. These analyses provided the explanation on the exclusive isolation of highly symmetrical *cis*-*cup*-conformer of tris(DPAF-C_9_). The fluorescent emission wavelength of *cis*-tris(DPAF-C_9_) was found in a close range to that of PVK with slightly lower HOMO (−4.81 eV) than that of Ir(ppy)_3_ (−4.9 eV). Therefore, this donor chromophore may serve as the secondary blue hole-transporting material, which is capable of minimizing the aggregation-related self-quenching effect, in the modulation of PLED performance. 

## Supplementary Materials

Supplementary materials can be accessed at: http://www.mdpi.com/1420-3049/20/03/4635/s1.
