# Supplementary material for: Synthesis and Photoluminescent Properties of Geometrically Hindered cis-Tris(diphenylaminofluorene) as Precursors to Light-Emitting Devices
Source: Molecules. 2015 Mar 13;20(3):4635–54. doi: 10.3390/molecules20034635 (PMC6272756; doi:10.3390/molecules20034635)
Supplement: Supplementary file 1 [file molecules-20-04635-s001.pdf]

Supplementary Materials

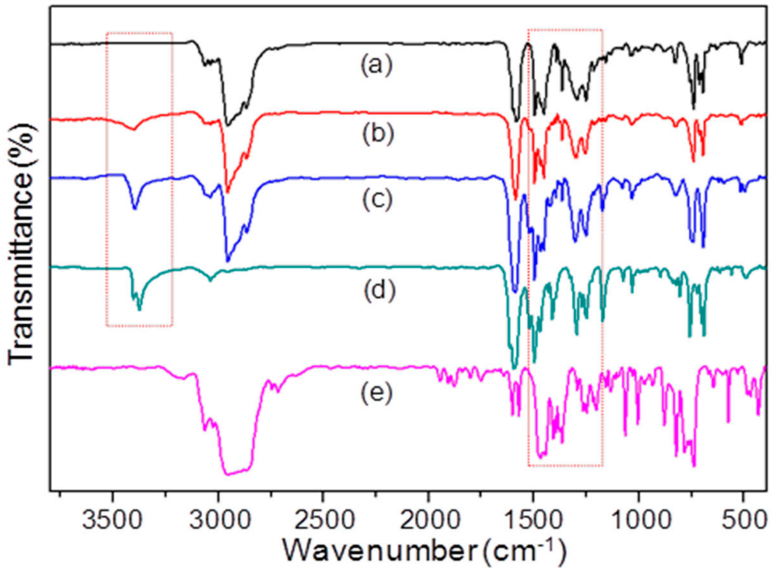

Figure S1. Infrared spectra of (a) tris(DPAF-C_9) **7**, (b) bis(DPAF-C_9) **6**, (c) mono(DPAF-C_9) **5**, (d) TPAB **4**, and (e) BrF-C_9 **2** collected on a KBr pellet.

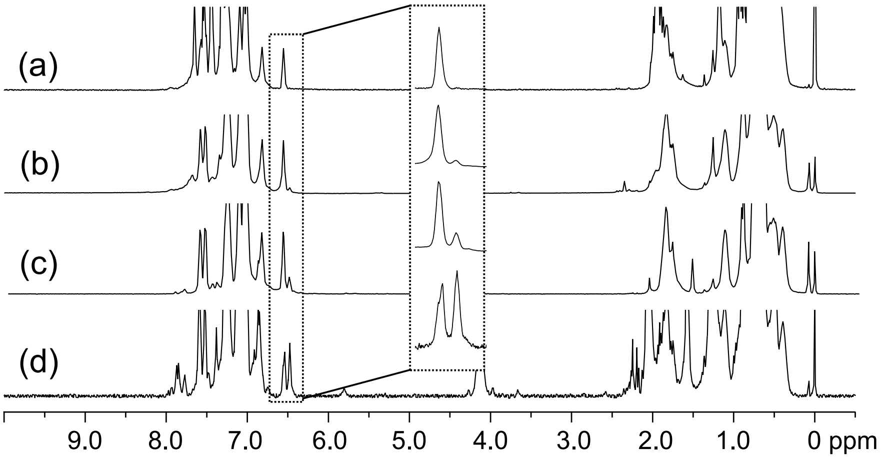

Figure S2. ^{1}H-NMR spectra of products using a different equivalent starting material ratio (TPAB/BF-C_9) of (a) 1:6 (excess), (b)1:4, (c) 1:3, and (d) 1:2.

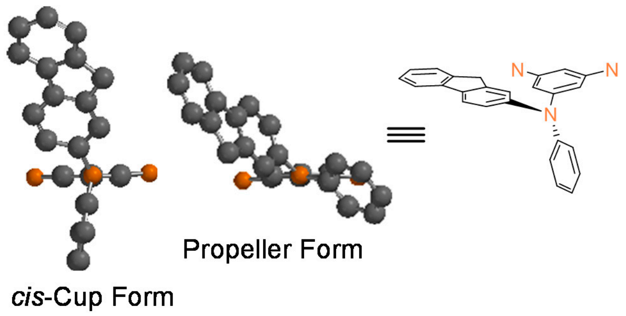

Figure S3. Torsional angle comparison between *cis*-cup and propeller forms showing much higher strain around the central triaminobenzene moiety. Other atoms are omitted for clarity.

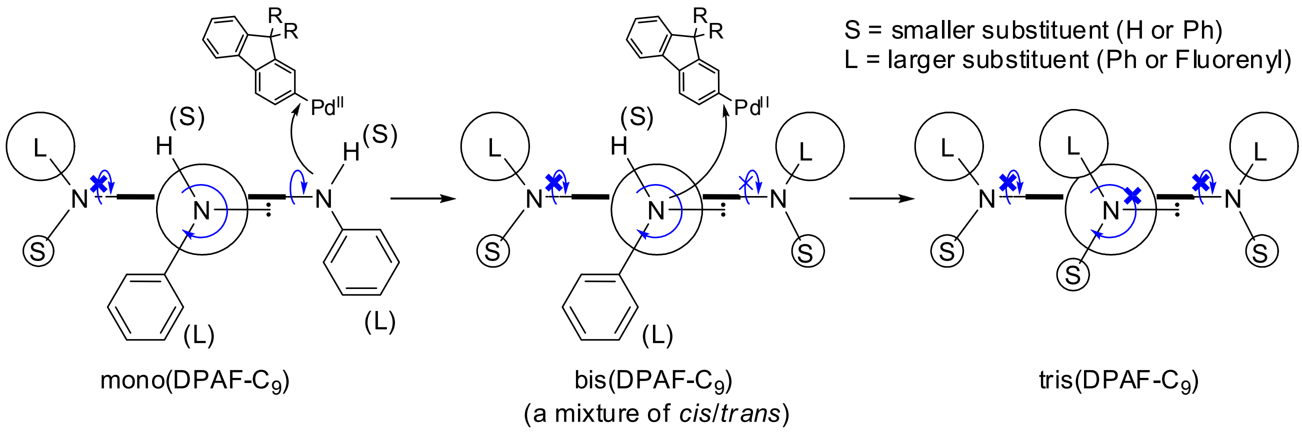

Figure S4. Alternative mechanism for the formation of exclusive *cis*-cup form by the restriction of central phenyl C-N bond rotation and the consideration of steric hindrance effect, leading to a preferred geometrical conformation going from mono(DPAF-C9) to tris(DPAF-C9) molecules.

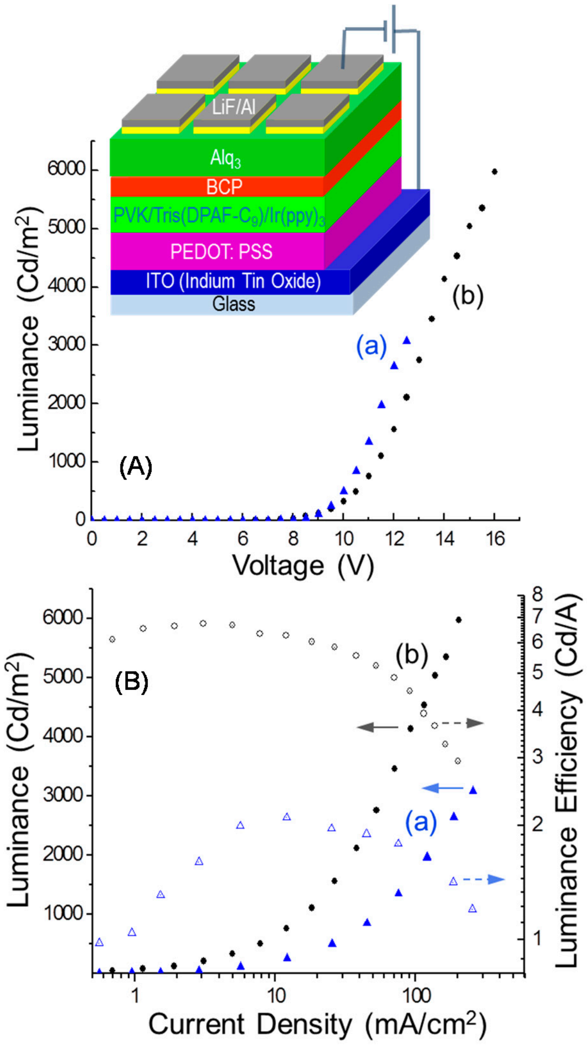

Figure S5. (**A**) Luminance (L)–voltage (V) and (**B**) luminance and luminance efficiency-current density (I) characteristics of multi-layered PVK–tris(DPAF-C9)–Ir(ppy)3 devices in a weight ratio of (a) 5:5:0.87 (▲/Δ) and (b) 10:0:0.87 (●/○).
